# Metaplastic LTP inhibition after LTD induction in CA1 hippocampal slices involves NMDA Receptor-mediated Neurosteroidogenesis

**DOI:** 10.1002/phy2.133

**Published:** 2013-10-24

**Authors:** Yukitoshi Izumi, Kazuko A O'Dell, Charles F Zorumski

**Affiliations:** 1 Departments of Psychiatry, Washington University School of MedicineSt. Louis, Missouri; 2 The Taylor Family Institute for Innovative Psychiatric Research, Washington University School of MedicineSt. Louis, Missouri; 3 Anatomy & Neurobiology, Washington University School of MedicineSt. Louis, Missouri

**Keywords:** Allopregnanolone, finasteride, long-term depression, metaplasticity, NMDA receptors

## Abstract

Long-term depression (LTD) induced by low-frequency electrical stimulation (LFS) in the CA1 region of the hippocampus is a form of synaptic plasticity thought to contribute to learning and memory and to the pathophysiology of neuropsychiatric disorders. In naïve hippocampal slices from juvenile rats, we previously found that LTD induction can impair subsequent induction of long-term potentiation (LTP) via a form of N-methyl-d-aspartate receptor (NMDAR)-dependent metaplasticity, and have recently observed that pharmacologically induced NMDAR-dependent LTP inhibition involves 5*α*-reduced neurosteroids that augment the actions of *γ*-aminobutyric acid (GABA). In this study, we found that both LFS-induced LTD and subsequent inhibition of LTP induction involve neurosteroid synthesis via NMDAR activation. Furthermore, the timing of 5*α*-reductase inhibition relative to LFS can dissociate effects on LTD and metaplastic LTP inhibition. These findings indicate that 5*α*-reduced neurosteroids play an important role in synaptic plasticity and synaptic modulation in the hippocampus.

## Introduction

N-methyl-d-aspartate receptors (NMDARs) play complex roles in synaptic function (Cull-Candy and Leszkiewicz 2004). Depending on how and when they are activated, NMDARs in the CA1 region of the hippocampus can induce long-term potentiation (LTP) or long-term depression (LTD). These forms of plasticity involve the influx of calcium through NMDAR channels and the activation of different intracellular signaling systems. In the case of LTP, a brief but high calcium influx results in preferential activation of protein kinases that drive early phases of synaptic enhancement (Malenka and Bear 2004). LTD appears to result from lower level, more protracted increases in calcium that initially activate protein phosphatases (Mulkey et al. 1993; Lisman 2001). Persistent plasticity involves changes in the trafficking of α-amino-3-hydroxy-5-methyl-4-isoxazolepropionic acid-type glutamate receptors, gene expression and protein synthesis (Malenka and Bear 2004).

NMDARs can also modulate the induction of synaptic plasticity. Certain patterns of synaptic activation (Coan et al. 1989; Huang et al. 1992) and mildly stressful conditions, such as brief hypoxia (Izumi et al. 1998) and low glucose (Izumi and Zorumski 1997), can result in NMDAR activation that results in no lasting effect on basal synaptic transmission, but inhibits subsequent LTP induction. These latter effects are referred to as “metaplasticity”, a form of synaptic modulation that alters the ability to induce other forms of plasticity (Abraham and Bear 1996; Abraham 2008). Although mechanisms contributing to NMDAR-mediated LTP inhibition are less defined than those contributing to LTP or LTD, our prior studies indicate a role for calcium (Izumi et al. 1992), calcineurin, and p38 mitogen-activated protein kinase (MAPK) (Izumi et al. 2008). Recent studies also indicate a role for NMDAR-stimulated synthesis of GABAergic neurosteroids, including allopregnanolone (alloP), in hippocampal pyramidal neurons (Tokuda et al. 2011). Several of these mechanisms are shared with LTD (Zorumski and Izumi 2012).

Prior studies also indicate that LTP induction can have a negative impact on subsequent LTD via effects on glycogen synthase kinase-3*β* (GSK3*β*) (Peineau et al. 2007), suggesting that the induction of both plasticity (LTP) and metaplasticity occurs with the same stimulation paradigm. Furthermore, abbreviated patterns of stimulation used for LTD (repeated 1 Hz stimulation) can prime synapses for LTD, making subsequent LTD induction easier (Mockett et al. 2002). It is presently unclear how and whether induction of LTD itself influences the mechanisms responsible for LTP and there is evidence that LTP and LTD can overcome each other's effects, leading to a situation in which synapses operate over a dynamic range of efficacy (Dudek and Bear 1992). In the present studies, we examined whether stimulation known to induce LTD has modulating effects on LTP induction at Schaffer collateral synapses in rat hippocampal slices and whether neurosteroids play a role in LTD and subsequent LTP modulation.

## Material and Methods

### Hippocampal slice preparation

Hippocampal slices were prepared from postnatal day (P) 30–32 albino rats using standard methods (Tokuda et al. 2010). Rats were anesthetized with isoflurane and decapitated. Dissected hippocampi were placed in ice-cold artificial cerebrospinal fluid (ACSF) containing (in mmol/L): 124 NaCl, 5 KCl, 2 MgSO_4_, 2 CaCl_2_, 1.25 NaH_2_PO_4_, 22 NaHCO_3_, 10 glucose, bubbled with 95% O_2_-5% CO_2_ at 4–6°C, and cut into 450 μm slices using a rotary slicer. Acutely prepared slices were placed in an incubation chamber containing gassed ACSF for at least 1 h at 30°C before further experimentation.

### Hippocampal slice physiology

At the time of study, slices were transferred individually to a submersion-recording chamber. Experiments were done at 30°C with continuous ACSF perfusion at 2 mL/min. Extracellular recordings were obtained from the apical dendritic layer (*stratum radiatum*) of the CA1 region for analysis of excitatory postsynaptic potentials (EPSPs) using electrodes filled with 2 mol/L NaCl (5–10 MΩ resistance).

EPSPs were evoked with 0.1 msec constant current pulses through a bipolar stimulating electrode in the Schaffer collateral (SC) pathway. A baseline (control) input–output curve was obtained to determine stimulus intensities for subsequent studies. Input–output curves were repeated 60 min after delivery of low-frequency stimulation (LFS; 1 Hz × 900 pulses), and then 60 min after delivery of high-frequency stimulation (HFS; 100 Hz × 1 sec) using stimuli of six different intensities to allow determination of how half maximal responses were altered. The smallest of these six stimuli was set to evoke a response less than half the maximum and the strongest stimulus was set to evoke fully saturated responses. The other four stimuli were delivered at equal intervals between these two stimuli. On the basis of analysis of input–output curves, we determined changes induced by LFS and HFS. During the course of an experiment, responses were monitored by applying single stimuli to the SC pathway every 60 sec at half maximal intensity. After establishing a stable control baseline for at least 10 min, LTD was induced by applying a 1 Hz stimulus to the SC pathway for 15 min. LTP was induced by a single 100 Hz × 1 sec tetanus using the same xlintensity stimulus.

### Immunohistochemistry

Studies examining changes in neurosteroid immunofluorescence were performed independent of electrophysiological studies. Slices used for immunohistochemistry were initially screened by electrophysiology and were incubated with various reagents in separate 10 mL beakers as previously described (Tokuda et al. 2010). Following LFS, slices were fixed in 4% paraformaldehyde in phosphate-buffered saline (PBS) for 30 min then washed with PBS and incubated in blocking solution (1% donkey serum/PBS) for 2 h at 25°C. Slices were incubated with an antibody raised in sheep against 5*α*-reduced neurosteroids diluted 1:2500 in 1% donkey serum/PBS for 48 h at 4°C then rinsed with PBS and incubated with secondary antibody for 2 h at 25°C. Alexa Flour 488 donkey anti-sheep IgG (diluted 1:500) was used to visualize neurosteroids. Confocal images were obtained using a 60× objective (1.4 N.A.), a C1 laser scanning confocal microscope and Z-C1 software (Nikon Instruments, Melville, NY). All parameters were kept constant within an experiment. Digital images were analyzed and the average intensity of the tissue was measured using MetaMorph software (Universal Imaging Corporation, Downingtown, PA).

### Chemicals

Finasteride and Alexa Flour 488 were purchased from Steraloids (Newport, RI) and Invitrogen (Carlsbad, CA), respectively. All other chemicals were purchased from Sigma (St. Louis, MO) or Tocris (St. Louis, MO). Anti-allopregnanolone antiserum was purchased from Dr. Robert Purdy, University of California-San Diego. Drugs were dissolved in ACSF at the time of experiment and administered by bath perfusion at the concentrations noted in the text. The concentrations selected for study and the durations of drug administration were based on prior studies indicating that the agents are effective at altering synaptic transmission or synaptic plasticity when administered in this fashion. Picrotoxin (PTXN) was dissolved in ethanol as a 10 mmol/L stock solution. When PTXN is dissolved in ethanol, concentrations of 0.5–1 μmol/L are sufficient to dampen GABA-mediated inhibition while avoiding the induction of marked epileptiform discharges.

### Statistical analysis

Data were collected and analyzed using PClamp software (Axon Instruments, Union city, CA). Data in the text are expressed as mean ± SEM 60 min following LFS or HFS, and are normalized with respect to initial baseline recordings (taken as 100%). A two-tailed Student's non-paired *t*-test was used for comparisons between groups. In cases of non-normally distributed data, the nonparametric Wilcoxon Rank Sum Test was used. Statistical comparisons were based on input–output curves at baseline and 60 min following 100 Hz or 1 Hz stimulation with *P* < 0.05 considered significant, and were done using commercial software (SigmaStat; Systat Software, Inc., Richmond City, CA).

## Results

**Figure 1:**
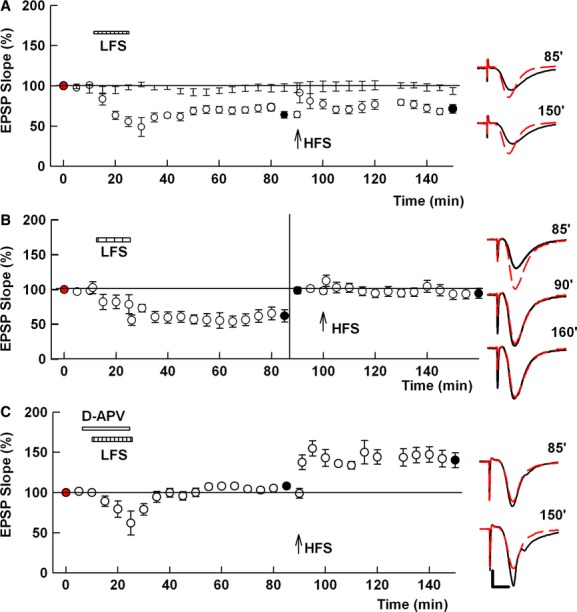
LFS induces LTD and LTP inhibition in the SC pathway. The graphs show the time course of change in EPSPs and traces to the right of the graph show EPSPs at the times denoted (black traces) with initial control responses shown as red dashed lines. (A) Following LTD induction by LFS (striped bar), HFS (arrow) failed to induce LTP. (B) As A, LFS and HFS were delivered in this order. After induction of LTD by LFS, however, the stimulus intensity was adjusted to evoke original degrees of EPSPs. HFS was delivered at this increased intensity. In spite of the augmented intensity, HFS failed to induce LTP. (C) In the presence of 50 μmol/L D-APV (white bar), LFS failed to induce LTD but LTP was successfully induced by subsequent HFS (arrow) after APV washout. Dots in A show base line response without any conditioning stimulations (*N* = 5). Traces to the right of the graph show EPSPs 60 min after LFS and 60 min after HFS at the times denoted with initial control responses shown as red dashed lines. Calibration: Calibration: 1 mV, 5 msec.

As shown previously (Dudek and Bear 1992), low-frequency stimulation (LFS) of the SC pathway at 1 Hz for 15 min (900 pulses) results in the induction of LTD (EPSP slope: 65.9 ± 5.9% of baseline 1 h after LFS, *N* = 6, Fig. 1A). Following stable induction of LTD for 1 h, subsequent administration of a 100 Hz × 1 sec high-frequency stimulus (HFS) to the same inputs failed to induce LTP (67.3 ± 5.7%, Fig. 1A). In naïve slices from P30 rats, this form of HFS reliably induces LTP (Izumi et al. 1992; Zorumski and Izumi 2012). After induction of LTD, half maximal responses are reduced. Thus, it is possible that the reduction in EPSPs alone is responsible for the failure of LTP induction by a subsequent HFS following LTD. In another set of experiments, we increased the intensity of the stimulus after LTD to evoke EPSPs similar to the original baseline prior to LTD (LTD = 66.7 ± 4.5%, *N* = 5). With the enhanced stimulus intensity, however, LTP was still not induced by HFS (99.9 ± 3.7% compared to the half maximal response taken 10 min prior to HFS; Fig. 1B). These results indicate that the failure of HFS to induce LTP after induction of LTD is unlikely to result from using a weak HFS. On the basis of prior results indicating a role for NMDARs in CA1 LTD (Dudek and Bear 1992; Izumi and Zorumski 2012) and metaplasticity (Abraham and Bear 1996; Zorumski and Izumi 2012), we examined the involvement of NMDARs using the broad-spectrum NMDAR antagonist, D-2-amino-5-phosphonovalerate (D-APV). Administration of 50 μmol/L D-APV, a concentration that totally suppresses NMDARs, before and during LFS blocked LTD (107.5 ± 3.2%, *N* = 6, Fig. 1C). Administration of D-APV during LFS also blocked metaplastic LTP inhibition and allowed a subsequent HFS to induce robust LTP (143.9 ± 9.2% 1 h after HFS, *P* < 0.001 vs. EPSPs 10 min before HFS, Fig. 1C). These results indicate that the activation of NMDARs during LFS drives LTD and also induces metaplastic LTP inhibition.

**Figure 2:**
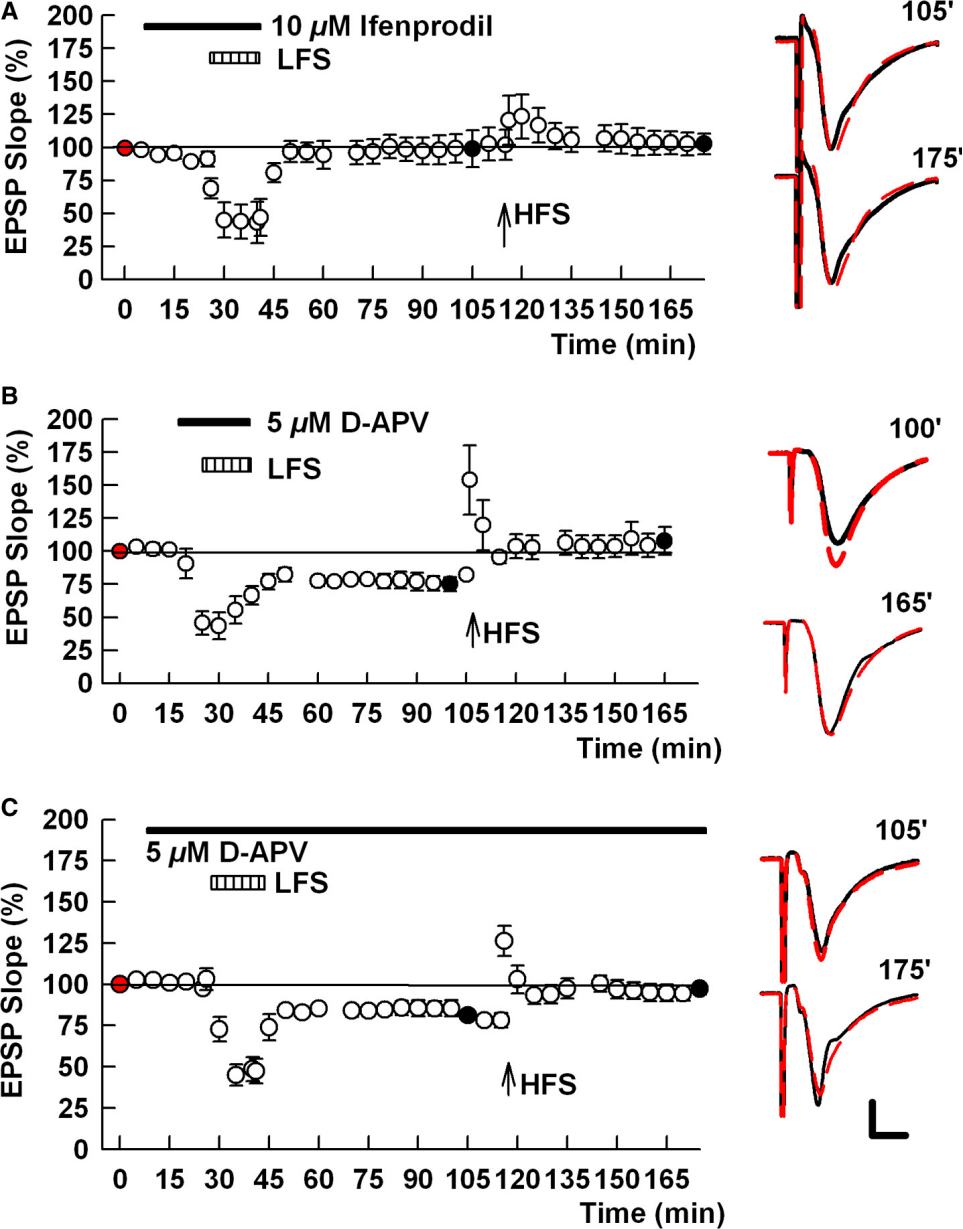
NR2B-containing NMDARs contribute to LTD but not to LTP inhibition. (A) Administration of 10 μmol/L ifenprodil (black bar), an NMDAR antagonist with selectivity for NR1-/NR2B-type receptors, completely blocks LFS (striped bar)-induced LTD but does not overcome LTP inhibition. (B) A low concentration of D-APV (5 μmol/L, black bar), administered during LFS does not alter LTD induction by LFS but overcomes LTP inhibition, allowing a subsequent HFS (arrow) to reverse LTD back to baseline. C. The effects of the briefer administration of 5 μmol/L APV is mimicked by a longer APV application throughout the experiment and shows no effect on LTD, but reversal of LTP inhibition. Traces to the right of the graph show EPSPs 60 min after LFS and 60 min after HFS at the times denoted with initial control responses shown as red dashed lines. Calibration: 1 mV, 5 msec.

There are several subtypes of NMDARs that are thought to contribute to synaptic plasticity (Cull-Candy and Leszkiewicz 2004). Previous studies have reported that the induction of LTD can be blocked by antagonists with selectivity for NR1-/NR2B-type receptors (Liu et al. 2004; Berberich et al. 2005; Izumi et al. 2005b, 2006; Bartlett et al. 2007), but that metaplastic LTP inhibition is not altered by NR2B antagonists (Izumi et al. 2006). Consistent with this, we found that 10 μmol/L ifenprodil, an antagonist with relative selectivity for NR1/NR2B completely blocked LTD (99.7 ± 3.5% 60 min after LFS, *N* = 5), but had no effect on subsequent LTP inhibition (99.6 ± 5.5% of baseline 60 min after HFS, Fig. 2A).

In contrast to ifenprodil, LTP inhibition can be prevented by low concentrations of APV (Izumi et al. 1992) that have preferential effects on NMDAR subtypes that are not blocked by pretreatment with ifenprodil (Izumi et al. 2006). Consistent with this, administration of 5 μmol/L D-APV just prior to and during LFS, did not inhibit LTD induction (79.9 ± 4.5%, *N* = 5) but allowed a subsequent HFS to potentiate depressed synapses back to original baseline levels prior to LTD (or allowed LTP induction if compared to the level before HFS) (102.1 ± 24.8% of original baseline EPSPs; 156.1 ± 38.5% if the half maximal response 10 min before HFS is set as 100%, Fig. 2B).

We also examined the effects of 5 μmol/L APV, continuously administered throughout the experiment including during the initial LFS and subsequent HFS, and found that this again failed to block LTD (73.8 ± 2.9%, *N* = 5) but allowed HFS to reverse depressed synaptic responses back to baseline (or allowed HFS to induce LTP) (92.3 ± 5.3%; 123.1 ± 3.8% if the half maximal response 10 min before HFS is set as 100%, Fig. 2C). Taken together, these results indicate that induction of NMDAR-dependent LTD has adverse effects on LTP and that the processes of LTD and metaplastic LTP inhibition can be differentiated pharmacologically.

**Figure 3:**
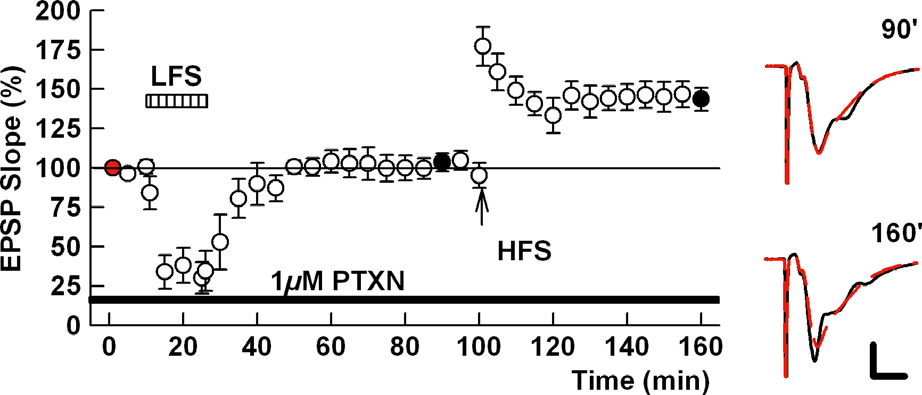
PTXN inhibits LTD induction. In the presence of 1 μmol/L PTX (filled bar), LFS (striped bar) failed to induce LTD but subsequent HFS could induce LTP. Traces to the right of the graph show EPSPs at the times denoted (black traces) with initial control responses shown as red dashed lines. Calibration: 1 mV, 5 msec.

In recent studies, we found that NMDAR activation associated with metaplastic LTP inhibition by low concentrations of NMDA promotes the synthesis of alloP and other 5*α*-reduced neurosteroids in CA1 pyramidal neurons and that these neurosteroids contribute to LTP inhibition (Tokuda et al. 2011). Because alloP is a potent positive modulator of GABA-A receptors (Akk et al. 2007), we initially examined whether PTXN, a GABA-A receptor antagonist, alters LTD. We observed that 1 μmol/L PTXN, administered continuously throughout an experiment, blocked the ability of LFS to induce LTD (104.0 ± 4.8%, *N* = 5; Fig. 3).

**Figure 4:**
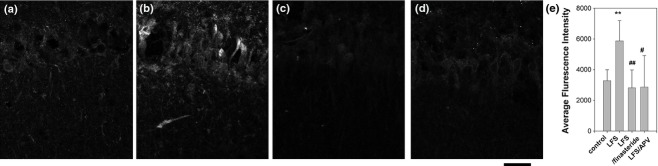
SC LFS increases neurosteroid immunoreactivity in CA1 pyramidal neurons. The photomicrographs depict immunostaining with an antibody against 5*α*-reduced neurosteroids in the CA1 region in a control slice (A), a slice exposed to 1 Hz × 15 min LFS (B), a slice exposed to LFS in the presence of 1 μmol/L finasteride (C) and a slice exposed to LFS in the presence of 50 μmol/L D-APV (D). Panel E shows a summary of neurosteroid immunostaining expressed in arbitrary fluorescence units. **P < 0.01 compared to controls; ^#^*P* < 0.05 and ^#^^#^*P* < 0.01 compared to LFS. Scale bar: 50 μm.

To pursue a role for neurosteroids in LFS-induced LTD and LTP inhibition, we examined whether LFS promotes neurosteroid production in the CA1 region. Using a previously characterized antibody against 5*α*-reduced neurosteroids, including alloP (Saalman et al. 2007; Tokuda et al. 2010), we found that 1 Hz × 900 pulse LFS increased neurosteroid staining in CA1 pyramidal neurons (Fluorescence Intensity: 3280 ± 290 in control slices and 5870 ± 540 in LFS-treated slices, *N* = 6 in each condition; Fig. 4) in a fashion that was inhibited by the NMDAR antagonist, APV (50 μmol/L) (Fluorescence Intensity: 2870 ± 1020). The increased staining by LFS was also blocked by finasteride (1 μmol/L), an inhibitor of 5*α* reductase, a key enzyme in neurosteroid biosynthesis (Fluorescence Intensity: 2810 ± 480; Fig. 4).

**Figure 5:**
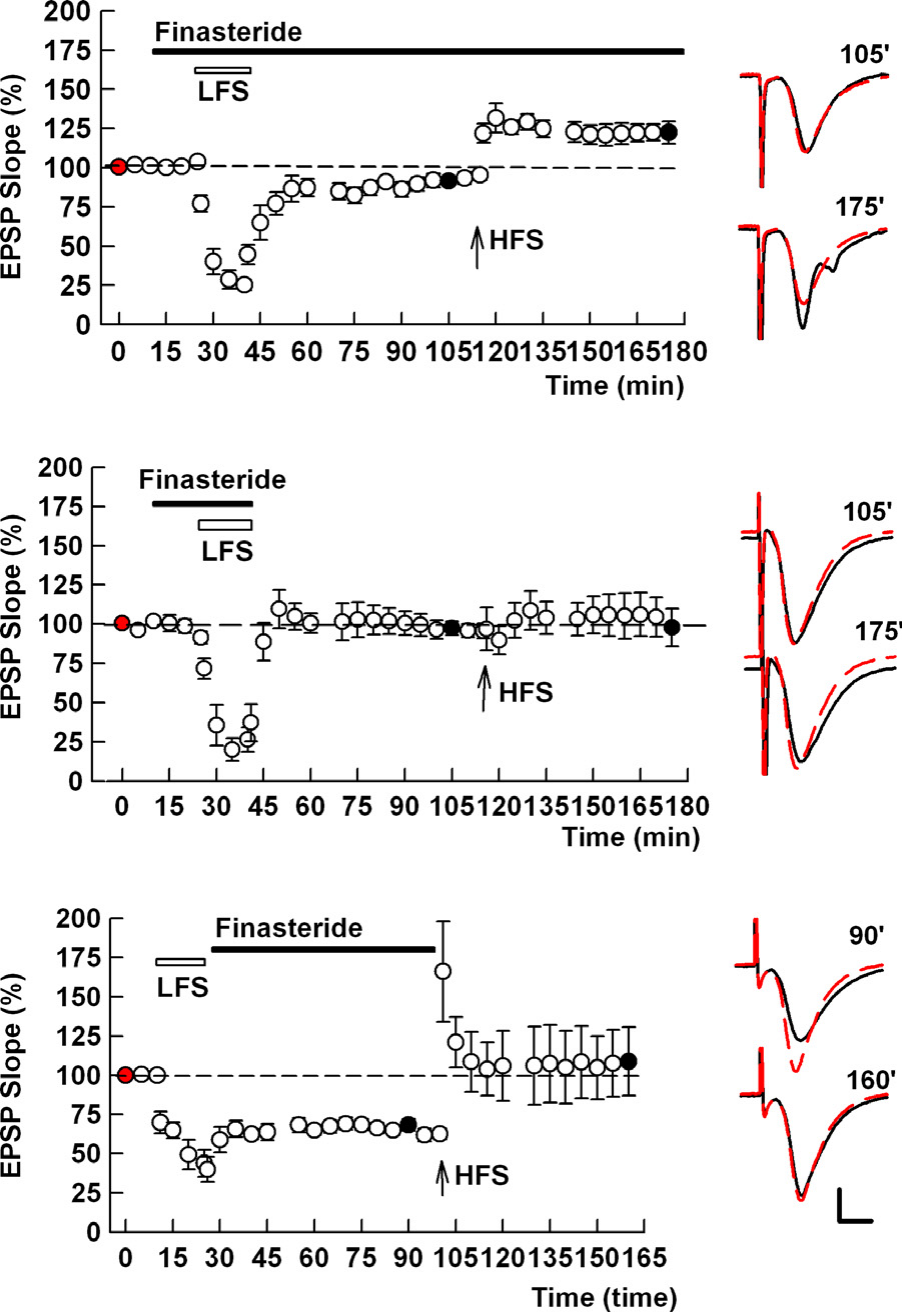
5*α*-Reduced neurosteroids participate in LTD and LTP inhibition. (A) Prolonged application of the 5*α*-reductase inhibitor, finasteride (1 μmol/L) during both LFS and HFS prevents homosynaptic LTD and LTP inhibition. (B) When administered for a shorter period prior to and during LFS, 1 μmol/L finasteride (black bar) blocks LTD induction but does not alter LTP inhibition. (C) Administration of finasteride immediately following LFS and continuing until delivery of HFS has no effect on LTD but allows HFS to reverse LTD back to initial baseline levels. Traces show representative EPSPs at the times denoted with initial control responses shown as red dashed lines. Calibration: 1 mV, 5 msec.

**Figure 6:**
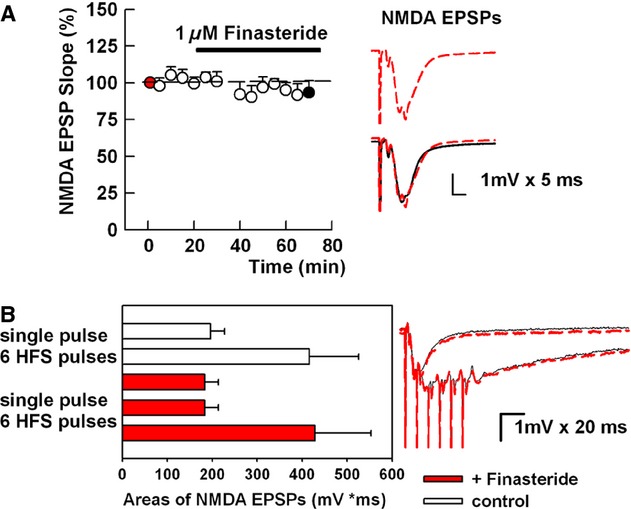
Finasteride does not alter NMDAR EPSPs. (A) In the presence of CNQX and low magnesium administration of 1 μmol/L finasteride (filled bar) has no effect on NMDAR EPSPs in the SC pathway elicited by a single puse. Traces show baseline EPSPs (red dashed top traces) with solid traces at the end of finasteride administration. (B) NMDAR-mediated EPSP areas were measured before and 30 min after administration of 1 μmol/L finasteride. NMDAR-mediated EPSPs were elicited by single pulse or by trains of six pulses at 100 Hz (open bars in histogram and solid traces are before and red bars and red dotted traces are 30 min after finasteride administration).

We subsequently examined the effects of finasteride on the ability of LFS to induce LTD and subsequent LTP inhibition. When administered continuously throughout an experiment, 1 μmol/L finasteride blocked both the induction of LTD and LTP inhibition (EPSP slope; 104.0 ± 12.0% 1 h after LFS; 134.2 ± 14.2% 1 h after HFS, *N* = 7 Fig. 5A). In order for finasteride to affect both LTD and LTP inhibition, the timing of administration was critical. When administered only prior to and during LFS, finasteride blocked LTD (97.8 ± 2.9%, *N* = 5) but had no effect on subsequent LTP inhibition induced by LFS 1 h later (91.6 ± 7.3%, Fig. 5B). In contrast, administration of finasteride immediately following LFS but during subsequent HFS had no effect on LTD (56.6 ± 4.8%, *N* = 5) but allowed HFS to reverse LTD (or allowed HFS to induce LTP if compared to levels before HFS) (101.9 ± 13.4%; 155.8 ± 27.5% if the half maximal response 10 min before HFS is set as 100%, Fig. 5C). Importantly, finasteride had no effect on NMDAR-mediated EPSPs, indicating that its effects do not result from NMDAR antagonism (Fig. 6A, 93.4 ± 8.1%, *N* = 3). Despite the lack of effect on isolated NMDAR EPSPs, it is possible that finasteride antagonizes NMDA-mediated responses during LFS (1 Hz) or HFS (100 Hz). In additional studies, we tested this by measuring the areas of NMDA-EPSPs evoked by brief trains of HFS. NMDA-EPSPs evoked by both single and six pulses of HFS were not altered by 30 min administration of 1 μmol/L finasteride (Fig. 6B). We also delivered six pulses of LFS (1 Hz) to evoke NMDA-EPSPs and compared the slope of NMDA-EPSPs. When the initial slope is normalized to 100%, the slope of EPSPs evoked by the sixth pulse was 84.3 ± 20.2% under control conditions. Thirty minutes after administration of 1 μmol/L finasteride, the slope of EPSPs evoked by the first pulse was 100.9 ± 13.3% of the control first NMDA-EPSPs, and the slope of NMDA-EPSPs evoked by the sixth pulse was 101.3 ± 15.9% of control (*N* = 5). These findings strongly suggest that finasteride is unlikely to depress NMDA-EPSPs even during HFS and LFS.

Table 1 presents a summary of results from experiments under the various conditions described above in which LFS was followed by HFS.

**Table 1:** Summary of LTD-LTP studies.

Condition (*N*)	LFS (%)	HFS (%)
Control (6)	65.9 ± 5.9	67.3 ± 5.7
50 μmol/L D-APV during LFS (6)	107.5 ± 3.2	143.9 ± 9.2
5 μmol/L D-APV during LFS (5)	79.9 ± 4.5	102.1 ± 24.8
5 μmol/L D-APV continuously (5)	73.8 ± 2.9	92.3 ± 5.3
1 μmol/L PTX continuously (5)	102.4 ± 2.8	166.4 ± 18.9
1 μmol/L Finasteride continuously (5)	104.1 ± 12.0	134.2 ± 14.2
1 μmol/L Finasteride during LFS (5)	97.8 ± 2.9	91.6 ± 7.3
1 μmol/L Finasteride after LFS (5)	56.6 ± 4.8	101.5 ± 13.4

## Discussion

The present results demonstrate that LFS-induced LTD is associated with metaplastic LTP inhibition in the SC pathway. In young slices, we observed that HFS delivered 1 h after LFS induction failed to induce LTP. Although previous reports show successful induction of LTP after LFS (Wexler and Stanton 1993; Wagner and Alger 1995), HFS was typically delivered shortly after LFS and/or in slices from mature rats in which LFS did not induce LTD. Both APV and finasteride block LTD and the subsequent LTP inhibition following LFS. We examined whether LTP inhibition requires LTD and found that both LTD and subsequent LTP inhibition require NMDAR activation during the initial LFS. However, it appears that LTD and subsequent LTP inhibition involves different subtypes of NMDARs, with LTD but not LTP inhibition being blocked by ifenprodil, a relatively selective antagonist of NR1-/NR2B-receptors (McCauley 2005). LTP, in contrast, is not sensitive to inhibitors of specific subtypes of NMDARs (Berberich et al. 2005; Izumi et al. 2006). Conversely, a low concentration of APV failed to alter LTD but overcame LTP inhibition. Although APV is a broad-spectrum NMDAR antagonist, prior studies have suggested that APV has some limited preference for NR1/NR2A type NMDARs (Buller et al. 1994). We previously observed that NMDA-EPSPs partially inhibited by ifenprodil were almost completely suppressed by addition of 5 μmol/L APV and that NMDA-EPSPs partially inhibited by 5 μmol/L APV were similarly suppressed by ifenprodil, suggesting that APV, at a low concentration, behaves like an antagonist of NMDARs that are insensitive to ifenprodil (Izumi et al. 2006). These results indicate that LFS induces LTD via activation of NR1-/NR2B-receptors, whereas LFS results in metaplastic LTP inhibition via activation of other, possibly NR1/NR2A, NMDAR subtypes. These results also indicate that metaplastic LTP inhibition associated with 1 Hz LFS does not require LTD. Although it is desirable to use antagonists with selectivity for NR1/NR2A receptors for further investigation, our previous and preliminary studies have failed to identify reliable and highly selective NR1/NR2A antagonists (Izumi et al. 2006).

The present results are consistent with prior studies indicating a role for NR1-/NR2B-type NMDARs in LTD (Liu et al. 2004; Massey et al. 2004; Berberich et al. 2005; Bartlett et al. 2007), although this has not been observed in all studies (Morishita et al. 2007). Previously, we found that NR2B-type receptors contribute to LTD in the CA1 region (Izumi et al. 2005b, 2006), but not to LTP inhibition produced by low concentrations of NMDAR agonists or chelation of extracellular zinc (Izumi et al. 2006). Prior studies have also shown that patterns of stimulation that lead to LTD can have positive metaplastic effects in which briefer trains of 1 Hz stimulation prime SC synapses for subsequent LTD induction (Mockett et al. 2002). Furthermore, we have found that low concentrations of NMDA (e.g., 1 μmol/L in 2 mmol/L Mg^2+^ × 5 min) are sufficient to inhibit LTP but do not produce LTD (Izumi et al. 1992), yet higher concentrations of NMDA result in a form of “chemical” LTD (Lee et al. 1998).

On the basis of recent work showing that 5*α*-reduced GABAergic neurosteroids are synthesized in CA1 pyramidal neurons (Saalman et al. 2007; Tokuda et al. 2010) and are involved in NMDAR-mediated LTP inhibition (Tokuda et al. 2011), we examined a possible role for neurosteroids in LTD and LFS-mediated LTP inhibition. These neurosteroids are synthesized from cholesterol by a series of steps that include the trafficking of cholesterol to mitochondria via steroid acute regulatory protein (STAR), translocation to the inner mitochondrial membrane via translocator protein 18 kDa (TSPO, also known as the peripheral benzodiazepine receptor), conversion into pregnenolone via P450 side-chain cleavage (SCC), trafficking out of mitochondria and conversion into alloP via 5*α* reductase and 3*α*-hydroxysteroid dehydrogenase (Chisari et al. 2010; Gunn et al. 2011). In this synthetic pathway, alloP is an end product and a potent positive modulator of GABA-A receptors (Akk et al. 2007; Gunn et al. 2011). Our interest in these neurosteroids was prompted by evidence that pyramidal neurons express the machinery for cholesterol homeostasis (Valdez et al. 2010) and are the principal cells in the CA1 region that express STAR (King et al. 2002), TSPO (Tokuda et al. 2010), the SCC enzyme (Kimoto et al. 2001) and 5*α* reductase (Agis-Balboa et al. 2006). Furthermore, NMDAR activation (Kimoto et al. 2001), including lower level NMDAR activation that results in LTP inhibition, enhances neurosteroid production in CA1 pyramidal neurons (Tokuda et al. 2011).

We found that both LTD and the metaplastic effects of LFS are blocked by finasteride, a 5*α* -reductase inhibitor. Interestingly, the timing of finasteride administration allowed dissociation of LTD and metaplasticity. When administered only prior to and during LFS, finasteride inhibited LTD but did not alter LTP inhibition. In contrast, administration of finasteride following LFS and during HFS allowed LTD induction but prevented LTP inhibition, whereas continuous administration throughout the recording period prevented both processes. Again, these findings support the idea that LTP inhibition does not require LTD. These findings further suggest that even though key events occur during LFS, enhanced neurosteroid production is likely an ongoing process triggered by NMDAR activation during the initial LFS.

How neurosteroids participate in LTD and LTP inhibition is uncertain, although the prominent effects of alloP on GABA-A receptors are leading possibilities (Akk et al. 2007; Gunn et al. 2011) and are consistent with the ability of PTXN to inhibit LTD (present study) and LTP inhibition (Zorumski and Izumi 2012). It is important to note, however, that prior studies suggest that neurosteroids play a necessary but not sufficient role in LTP inhibition. That is, administration of exogenous alloP alone is insufficient to inhibit LTP or to induce LTD at concentrations up to 1 μmol/L (Izumi et al. 2007; Tokuda et al. 2010). Rather, alloP appears to work in concert with other mechanisms to dampen LTP induction. For example, in the case of the amnesic benzodiazepine, midazolam, effects on LTP and learning require activation of both TSPO and the flumazenil-sensitive benzodiazepine site on GABA-A receptors (Tokuda et al. 2010). Neither effect alone is sufficient to inhibit LTP. Whether neurosteroids play a similar permissive role in LTD by interacting with other mechanisms involved in LTD and LTP inhibition remains to be determined.

Our results have implications for the roles of LTP and LTD in learning and memory. Both forms of plasticity are thought to contribute to specific forms of learning and to hippocampal network function (Martin et al.^2000^; Kemp and Manahan-Vaughan 2007). Furthermore, metaplastic LTP inhibition appears to be relevant to a variety of stressful conditions, including brief hypoxia (Izumi et al. 1998), low glucose (Izumi and Zorumski 1997), ammonia (Izumi et al. 2005a, 2013), and acute behavioral stress (Kim et al. 1996; Yang et al. 2008). Thus, this form of metaplasticity could be relevant to learning defects observed in a range of neuropsychiatric disorders and could provide novel approaches for cognitive enhancement.
